# A Distributed Multi-Tier Emergency Alerting System Exploiting Sensors-Based Event Detection to Support Smart City Applications

**DOI:** 10.3390/s20010170

**Published:** 2019-12-27

**Authors:** Daniel G. Costa, Francisco Vasques, Paulo Portugal, Ana Aguiar

**Affiliations:** 1 Department of Technology, State University of Feira de Santana, Feira de Santana 44036-900, Brazil; 2 INEGI/INESC-TEC—Faculty of Engineering, University of Porto, 4200-465 Porto, Portugal; vasques@fe.up.pt (F.V.); pportugal@fe.up.pt (P.P.); 3 Instituto de Telecomunicações (IT), University of Porto, 4200-465 Porto, Portugal

**Keywords:** emergency alerting, event detection, sensors monitoring, Internet of Things, smart cities

## Abstract

The development of efficient sensing technologies and the maturation of the Internet of Things (IoT) paradigm and related protocols have considerably fostered the expansion of sensor-based monitoring applications. A great number of those applications has been developed to monitor a set of information for better perception of the environment, with some of them being dedicated to identifying emergency situations. Current IoT-based emergency systems have limitations when considering the broader scope of smart cities, exploiting one or just a few monitoring variables or even allocating high computational burden to regular sensor nodes. In this context, we propose a distributed multi-tier emergency alerting system built around a number of sensor-based event detection units, providing real-time georeferenced information about the occurrence of critical events, while taking as input a configurable number of different scalar sensors and GPS data. The proposed system could then be used to detect and to deliver emergency alarms, which are computed based on the detected events, the previously known risk level of the affected areas and temporal information. Doing so, modularized and flexible perceptions of critical events are provided, according to the particularities of each considered smart city scenario. Besides implementing the proposed system in open-source electronic platforms, we also created a real-time visualization application to dynamically display emergency alarms on a map, demonstrating a feasible and useful application of the system as a supporting service. Therefore, this innovative approach and its corresponding physical implementation can bring valuable results for smart cities, potentially supporting the development of adaptive IoT-based emergency-aware applications.

## 1. Introduction

The advent of more efficient and cheaper technologies for distributed data acquisition has paved the way for new types of applications, putting the machine-to-machine IoT-centric paradigm in use [1,2]. When it was advocated that some frequent urban problems should be mitigated by the evolving IoT concept, some initiatives around the creation of smart cities took place [3]. Currently, a considerable number of monitoring applications is already in operation in many large cities, providing perceptions about people, animals, vehicles, buildings, weather or any measurable variable through the acquisition of different types of sensed data [4].

Urban areas may provide a huge amount of information retrieved from deployed sensor nodes, vehicles or even their inhabitants, which can be realized by the great number or current applications employing sensor networks [2], smartphone-based crowdsensing [5] or even social media mining [6]. Such sensed data can be used for many functions, including emergency detection and alerting. An emergency is a situation that can result in physical injury to people and also cause economic losses, mostly happening in an unpredictable way [7,8]. Therefore, finding an efficient way to manage emergencies in these contexts is of paramount relevance.

Some sensor-based applications have been developed to alert people during the occurrence of an emergency, but focus on a single phenomenon, for example in fire [9] and flood [10] detection. While such applications may be efficient in particular cases, they are limited in the sense that they do not adapt to different scenarios. Moreover, when employing individualized sensing networks for emergency detection and alerting, different independent systems might be required to alert people during multiple emergency situations. With the large scale integration expected for smart cities, and with IoT-based systems operating to provide ubiquitous integrated services [11], the way emergencies are detected and reported should be improved, fostering the development of this work.

An emergency may have different levels of severity depending on its potential of damage, its area of effect and its temporal significance, resulting in different cities and neighborhoods perceiving an emergency situation in different ways. Whatever the case, we can trace a direct line between an emergency and the occurrence of critical events, since an event must be detected in order to trigger an emergency alarm. While there may be different ways to detect or even to value an event [12], we define that any event is an ON/OFF situation that has to be properly detected and associated to a GPS coordinate, and thus each event is assumed as detected (ON) or not detected (OFF) in a defined instant of time. Afterwards, a detected event will trigger an emergency alarm, but its “magnitude” will depend on different personalized configurations. This separation of “critical events” and “emergency alarms” as two related but different concepts is one of the contributions of this work.

A practical example of the proposed separation of those concepts is described as follows. The variable “fire” may be measured by a temperature sensor, which provides information within a range of possible values (e.g., in Celsius). A temperature threshold may then be defined for the retrieved data from that sensor, for example establishing that a critical fire event is detected (ON) when it is equal to or higher than $80^{\circ }$. After that, emergency alarms with different levels of severity may be created for exactly the same event: For neighborhoods with many wooden houses, an emergency alarm should be more critical than if the same event was detected on a sparsely populated area. Therefore, the proper definition of how events will be detected and how emergency notifications (alarms) will be created and delivered are central for the proposed emergency alerting system.

In order to perform efficient, adaptive and highly configurable emergency alerting in heterogeneous smart cities, we propose a modularized system to continuously generate emergency alarms (EAs) responsively. For that, the proposed system is divided into three logical tiers: Events, alarms and applications. As an emergency alarm may only be created for the occurrence of at least one critical event, a number of event detection units (EDUs) are expected to be distributed over a region, detecting events that are associated to a GPS-based position. The tier events will then be composed of one or more EDUs, which will transmit event reports (ERs) to an emergencies processor unit (EPU). The EPU is the logical unit of the tier alarms and it is ultimately responsible for creating and transmitting emergency alarms to all registered applications, emergency alarms clients (EACs), in a broker-oriented architecture. The tier application contains then all EACs that want to receive emergency alarms from the system. This multi-tier logical operation assures flexibility and scalability during the entire operation cycle of emergency alerting.

**Figure 1:**
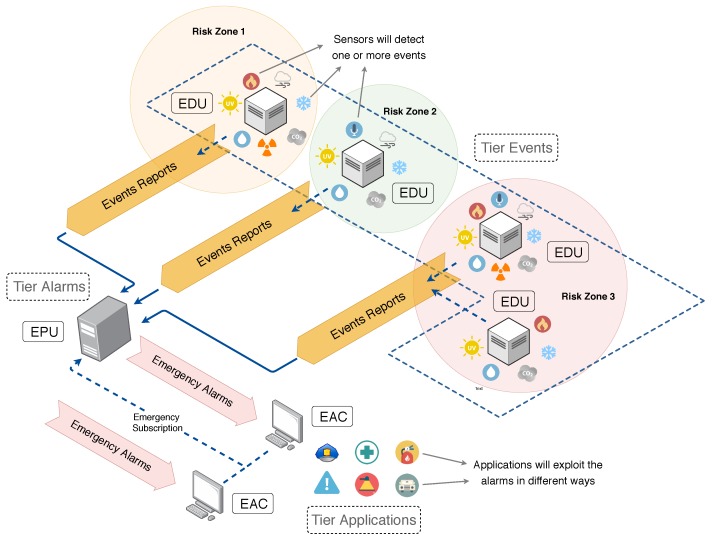
The three logical units of the proposed system and the communication flow between them.

Figure 1 presents a schematic operation of the proposed system, referred as CityAlarm, highlighting the theoretical flow of messages and the communication between the units. The operation and implementation details of all presented units will be described in next sections.

In order to make the proposed system fully available for practical use and experimentation, we implemented both the EDU and the EPU units in the open-source Raspberry Pi platform [13], making both units affordable and able to be easily reproduced. Additionally, we developed a web-based application for visualization of emergency alerts, which were simulated in the city of Porto, Portugal. The idea is to allow users to graphically view emergency alarms on a real-time basis, plotted on a map. The definitions of the proposed system along with the provided implementations, which are freely and openly available for usage and modification, are important contributions for practical emergency alerting in smart cities.

Finally, the proposed system provides integrated emergency alerting as a supportive tool for any requesting application, in a different way to previous works. This is accomplished through a series of contributions, summarized as follows:

1. Distributed detection and alerting of emergency situations through a multi-tier architecture.
2. Separation of critical events and emergency alarms, which are processed individually and by different logical units.
3. Definition of critical events as logical (ON/OFF) parameters, while emergency alarms are defined as having a numerical magnitude within a considered range.
4. Computation of the impact of emergency alarms based on three distinct elements: Number of detected events, spatial information (modelled as “risk zones”) and temporal significance.
5. Delivery of emergency alarms in a distributed and flexible way, attending any requesting application. The alarms are delivery in an open standard format.
6. A reference implementation of all logical units, which was created for the Raspberry Pi platform and is available at https://github.com/lablara/cityalarm.git.

The remainder of this article is organized as follows. Section 2 describes relevant works in the literature that influenced this work and that highlight the urgency of automatic effective emergency alerting. The fundamentals of the proposed emergency alerting system are presented in Section 3. Section 4 introduces the event detection unit (EDU), describing the adopted approach for event detection. The definitions of the emergencies processor unit are described in Section 5. Then, Section 6 presents a proof-of-concept of CityAlarm and also a web-based application to graphically exhibit reported emergencies. Section 7 discusses smart city perspectives of the proposed approach, pointing out potential applications. Finally, conclusions and references are presented.

## 2. Related Works

The development of monitoring applications in smart cities is fast paced, with designing, development and operation challenges being constantly addressed by different strategies and methodologies. In this evolving scenario, different solutions for IoT and particularly smart cities have been proposed, influencing this work in different ways.

Among the objectives of typical monitoring applications for the scope of smart cities, emergency detection, alerting and response have received considerable attention in the last decade [14]. Such applications have addressed different aspects of these “services”, mainly due to the fact that emergency alerting is a very complex task, especially for large scale scenarios as in smart cities. Their achieved results are then important clues for the construction of more efficient and flexible IoT-based emergency management systems.

When reviewing the literature concerning emergency detection and alerting, some works have provided practical solutions from different perspectives. In fact, automatic solutions for emergency detection are not a novelty, even when we consider the adoption of sensor-based monitoring systems. Therefore, the state-of-the-art in this area has presented solutions for different challenges of this problem and there are many works bringing some contributions in this sense.

Concerning the variety of contributions for emergency detection and alerting, we identified four different attributes that are core characteristics of the proposed CityAlarm system, but that were addressed in different ways in the literature. Those characteristics are discussed as follows:

- Monitoring scope: How many emergency situations can be detected?
  – Previous works: Several research and development works have proposed solutions to detect a single or a limited number of emergency situations, since a lot of systems have been developed around a pre-defined number of sensor types. For example, the work in [15] exploited sensor nodes to detect flood in homes, raising an alert when there is a water leak. For that, a sensor device was designed to detect a critical flood emergency. While such works can operate efficiently, they have limited application and a smart city scenario may need to deploy a group of different systems to accomplish a comprehensive and unified emergency detection solution.
  – Proposed approach: As the proposed CityAlarm solution is a multi-tier emergency detection and alerting system designed to handle any type of emergency situation, it can be considered as a unique solution for an entire city, potentially avoiding compatibility and inter-operation problems that may arise when multiple different systems are concurrently operating.
- Emergencies’ significance: What is the impact of detected emergencies?
  – Previous works: There has not been a consensus on the significance of detected emergency situations in the literature, with different approaches giving different perceptions of it. The work in [16] employed a wireless sensor network to alert people to a fire emergency in smart homes in a quick and effective way. Employing temperature, gas and smoke sensors to increase effectiveness and confidence, this approach is efficient when detecting a “fire” emergency. However, an emergency according to [16] is an ON/OFF variable and all detected emergencies are the same, regardless of where or when they happen. Nevertheless, an emergency can also be alerted providing additional information, as in [17]. In that work, sensors are used to alert people when the pollution level is above a defined threshold, but the level of pollution is also made clear. A lot of works have proposed different solutions when alerting to emergencies, which may be performed with the provision of different types of information.
  – Proposed approach: The CityAlarm system extends the perceptions of emergencies’ significance, associating three different groups of information: Number of detected events, risk area and temporal relevance. This is possible because CityAlarm is not dedicated to addressing a particular emergency (flooding, fire, natural disaster, etc), but any possible emergency that can be measured using sensor devices. Additionally, the concepts of “events” and “emergencies” are formally separated in CityAlarm, which allows the addition of new elements to the perception of emergencies. In this particular case, the perception of critical events and the associated definition of emergencies have been frequently coupled in emergency management systems [18,19]. In a different way, the detected emergencies in CityAlarm are associated with a numerical value within a range and such a value is computed based on those three groups of information. As a consequence, the proposed approach becomes more flexible and comprehensive in being adopted in any emergency detection and alerting scenario.
- Configuration of the system: How easy is its configuration and re-configuration?
  – Previous works: This characteristic varies considerably, but it is reasonable to say that most systems can be easily configured when properly defined for that purpose. The limitations that may arise then do not result from their configurations, but from the expected services of the systems. For example, if a fire detection system is designed, it is expected to be easily configured concerning such variables (thresholds, detection range, messages recipients, etc), but other variables (such as noise and pollution) may not be easily configurable (or not configurable at all). The configuration of those systems has been facilitated with the adoption of flexible open-source hardware platforms, such as Raspberry Pi and Arduino, a recent trend that influenced CityAlarm. In [20], the authors proposed a rainfall emergency notification solution to alert people to critical situations, using the Raspberry Pi platform to detect them. Besides sensing the rain level, the created nodes can perform predictions about upcoming heavy rain, which could be easily reconfigured or adapted for different scenarios.
  – Proposed approach: In order to adapt to the particularities of the different monitoring scenarios, the CityAlarm system was designed to be easily configured and re-configured, following a trend found in the literature. The proposed system is modularized for better adaptation to different monitoring scopes, making it easy to define events of interest, emergency alarms and risk zones. Moreover, the JSON format was adopted for the creation of the system’s messages, this concept being borrowed from web-based applications. Doing so, the messages transmitted between the CityAlarm units cannot only be easily changed, but it also facilitates the processing of the messages by other systems. Finally, the development of a proof-of-concept based on the Raspberry Pi platform follows a recent trend in the literature [13,21], facilitating the development of practical applications based on the proposed system.
- Scalability: How scalable is the emergency detection and alerting system?
  – Previous works: Scalability is a desired characteristic for some scenarios, especially for modern large cities. However, it may be hard to achieve in many cases. For systems dedicated to addressing a specific emergency situation in a defined scenario, for example fire detection in a residence[16], scalability may not be a concern, as such systems will operate individually. However, some solutions may cover large areas that may even change during the operation of the system. For example, the work in [22] defines a wireless sensor network to collect information about rainfall in different places and, using the cellular network, eventually alerting people to heavy rainfall as an emergency. In such a system, scalability may be a concern when many sensor units are deployed and have to deliver real-time information. In the literature, scalability issues have guided the development of some systems for emergency detection, usually defining multiple logical tiers for detection of critical situations and processing of alarms [23,24], influencing the development of CityAlarm.
  – Proposed approach: Having scalability as a fundamental characteristic, the proposed system was designed to detect, to process and to alert people to emergencies through different logical tiers, this concept being borrowed from recent works on IoT-based monitoring. Moreover, the separation of the concepts of “events” and “emergencies”, which are processed in different logical tiers, also reinforces the scalable nature of the proposed system. The expected scalability of the proposed system needs to be properly evaluated, but the modularized operation of the system following some recent trends is a good indication in this direction.

As could be seen, the proposed system borrows some concepts from recent works in the literature, leveraging good ideas and concepts when providing emergency detection and alerting. Moreover, the limitations of many existing works, notably on the detection of only particular emergencies and the delivery of non-comprehensive emergency alarms, were addressed by CityAlarm, which comes as a unified and supportive solution for emergency-oriented applications in smart cities.

While much research efforts have been dedicated to perform early detection of emergency situations, as previously discussed, other relevant matter is the action to be taken after an event is properly detected. When an event is detected and an emergency situation is assembled (as discussed in Section 4 and Section 5), an emergency alarm must be issued. In this sense, we define that an “emergency alarm” is a generic indication of an active emergency situation, comprising sufficient meta-data to support any possible action, for example a textual warning or the triggering of an actuator. Considering the literature in this area, emergency alarms have been processed in four different ways:

- Warning messages: This is the most obvious form of notification. When a critical event is detected and an emergency alarm is assembled, systems may send different types of messages to alert users about what is happening and possibly the level of criticality of the emergency. Such an alert may be a textual message, an audio warning or even an informative video. For example, users are alerted by GSM (global system for mobile communication) messages transmitted to their smartphones in [16]. In a different way, in the work presented in [15], a siren was used for audio alerting, besides textual messages.
- Rescue and exit paths: When using complementary information, an emergency alarm can be exploited to define rescue and evacuation paths for people in danger. For the work in [25], an analytical model was defined to compute the fastest escape route when an emergency is signalled. For that work, different information is crossed to achieve the most efficient route for people in danger. In [26], sensor nodes were used to identify safe exit paths during an emergency situation since sensors may be in a hazard or non-hazard mode according to the detection of a previously expected critical event. In both cases, users interact with the system through the definition of exit paths.
- Actuation and counter-measures: Some works have focused on acting to solve a particular emergency situation by removing its causes, resulting in a class of applications for disaster recovery. In such cases, an emergency alarm is an input for a recovery (concurrent) system, driving some counter-measure. In [27], detected emergencies were exploited to trigger mobile actuators, which are moved toward the sensors that detected the related events. Such actuator nodes may then gather additional information of the emergency or even do something to eliminate the cause of the detected events. In [28], sensor nodes were used to detect dangerous gas. When gas is detected, actuators are employed to isolate the gas leak source through the manipulation of gas valves. In both cases, the actuator system is complementary and concurrent to the emergency detection solution.
- Configuration of systems: An emergency alarm may also be realized as a configuration parameter for concurrent systems. Exploiting this type of information, a system can operate due to simple configurations, such as switching a sensor node from idle to active mode, or even determining how sensors will gather and process information from the environment. In [29], critical events were detected by sensor nodes, which exploited such information to configure their own sensing operation (transmission pattern, visual data sampling, media coding and QoS-based routing) during the time the event was still assumed as detected. The idea is to increase the level of details of retrieved data during an emergency, even consuming more energy and resources if necessary. The work in [30] considered the occurrence of critical events to change how logical clusters are created in the Medium Access Control (MAC) layer for more efficient communications during an emergency. QoS-based prioritization of sensor nodes has been addressed in recent years, it being a promising “client” for emergency management systems [12].

There are different ways to process an emergency alarm and the literature presents some promising examples in this area. However, all previous works have limited applicability in smart cities. We believe that an emergency alerting system for large-scale smart city environments should be flexible enough to support different approaches when emergency alarms are released, allowing any processing of the received alarms. However, no work in the literature has addressed all the presented issues. The CityAlarm comes then as an efficient, generic, flexible and distributed emergency alerting system that is devoted to supporting emergency-oriented applications, bringing an important and novel contribution to the area.

## 3. The Fundamentals of the Proposed Emergency Alerting System

The proposed system is expected to detect critical events in a distributed way, processing such events along with other relevant information to generate georeferenced emergency alarms. Some fundamental concepts have to be defined, as presented in this section.

### 3.1. Concepts and Basic Definitions

As the proposed system will handle a good deal of different information from the conceptual logical units, it is worth to formally define all concepts and elements related to the system. Such basic definitions are expressed as follows:

- Event of interest (EI): This is a critical “situation” that may put people in danger or may result in some relevant economic losses. We define that any event is directly mapped to a single “type” of sensed data that can be represented within a numeric scale. Thus, an EI will be associated to a single variable that can be measured by a particular sensor device, such as temperature, humidity, pressure, noise and luminosity, among others.
- Event report (ER): This is a description message indicating the types of all detected events of interest, along with the GPS coordinates where those events are detected and a timestamp mark. The event reports are created every time there is at least one change in the current list of detected events, in a hybrid synchronous/asynchronous way (described in Section 4), being transmitted toward the emergency processing unit. Every event report results in a single emergency alarm.
- Risk zone (RZ): When employing the CityAlarm system, the considered application area is expected to be divided into one or more risk zones. For simplification purposes, a risk zone is defined by a circular area and is associated to a numerical risk level. The association of a risk level to each RZ is a subjective decision that should take into account the risk factors for disasters, such as the number of people in the area and the presence of rescue and emergency response infrastructure. Each risk zone may contain zero or more event detection units.
- Emergency alarm (EA): This is the expected outcome of the CityAlarm system. An emergency alarm will be generated for each event report individually. Therefore, every EA message will contain the indication (“types”) of all events of interest presented in the ER. Moreover, the GPS coordinates (provided by the ER) will also be carried on emergency alarms, since they are required to determine the corresponding risk zone associated with the detected events, and applications may exploit such information. Together with the ER timestamp, a severity level (magnitude of the emergency) is also computed and inserted into the EA. All of this information can then guide different emergency actions, as reviewed in Section 2.
- emergency subscription (ES): This is a generic process performed by an external application that wants to receive emergency alarms from CityAlarm. While such a request may pass through some authentication process, it is out of the scope of this work, only being suggested in the case where emergency alarms are considered as confidential information. Nevertheless, the emergency subscriptions are expected to operate considering a broker-oriented architecture (described in Section 5).

These five concepts are the core elements of the proposed emergency alerting system.

### 3.2. Processing and Communication Units

The CityAlarm system is composed of three logical units that communicate among themselves to achieve the expected results following a well-defined communication flow. Actually, each of these units corresponds to a conceptual tier that is responsible for a group of tasks in the operation cycle of the proposed emergency alerting system. The definitions of these units are presented as follows:

- Tier events—event detection unit (EDU): This unit is the fundamental block of the CityAlarm system since it is responsible for the detection of events of interest, which are the defining conditions for an emergency alarm. An EDU is a multi-sensor unit that is capable of detecting as many different EI as there are sensor devices attached to it. Additionally, an EDU should be attached to a GPS device, allowing it to know its current location, but a cheaper implementation could manually configure the GPS coordinates into each EDU when setting them up. The GPS coordinates are required for the event reports that are transmitted by the EDU, indirectly matching any EDU to a risk zone. However, the concept of risk zone is unknown by the event detection units, being employed only by the EPU. Typically, there should be many EDUs spread over a smart city, increasing the precision and reach when detecting emergency situations;
- Tier alarms—emergency processing unit (EPU): This unit is responsible for receiving all event reports and processing them considering all defined risk zones (which are manually configured according to characteristics of the considered city) and the time scope configurations (also dependent on the considered city). Doing so, emergency alarms are generated in an adaptive and configurable way, which is more realistic and useful for modern cities. For that, every EDU must know the current location of the EPU: The number of emergency processing units may vary according to the size of the city and the expected effectiveness of the emergency alerting system, but we assume that at least one EPU will be deployed. Besides the constant transmissions from one or more EDUs, the EPU will also (indirectly) interact with the emergency alarm clients, who are the final users of the system;
- Tier applications—emergency alarm clients (EACs): Emergency alarms are generated to be exploited by any emergency alarm client. Initially, an EAC must subscribe to an EPU, in a direct or indirect way (described in Section 5), using a standard registration process. After that, an EAC must keep waiting for emergency alarms. The exploiting of the emergency alarms by the EACs is out of the scope of the proposed system, but some common applications are discussed in Section 6.

Considering a networking perspective, the CityAlarm is an application-layer system and thus any networking technology and paradigm can be employed. For implementation purposes, the EDU, the EPU and the EACs are implemented using the TCP/IP protocol stack, but even basic addressing can be easily adapted if required, without compromising the proposed approach.

## 4. Detecting Events of Interest

The proposed emergency alerting system is designed to exploit the concept of events of interest (EI), as mentioned earlier. Therefore, event detection is a fundamental part of CityAlarm. Since the concepts of “events” and “emergencies” are not coupled in this work, the detection of events must be properly defined and implemented, as described in this section.

### 4.1. Defining an EI

In short, an event of interest is any critical “situation” that is relevant enough to be considered since it has a potential to hurt people or cause relevant economical losses. In fact, it has to be highlighted that an EI has a “potential” to cause damage, but the actual damage is not estimated or assessed by CityAlarm. Nevertheless, such potentiality cannot be neglected, and thus a detected event of interest will always result in an emergency alarm.

We define that an event of interest is directly mapped to a single “type” of scalar sensed data, although the same sensor device may provide data to trigger more than one different EI. In this sense, an event report is detected through a physical sensor of temperature, while an event of low humidity is detected through a sensor of humidity, and both events may be independently detected by the same EDU, at the same time. Thus, the (single) emergency alarm resulting from these events of interest will inform that it was caused by events of heating and low humidity, which may indicate that a fire emergency is happening (or just a generic emergency that demands a state of “caution”).

A fundamental question that arises when detecting events is how to identify what is “critical”. Actually, different contexts may define different perceptions of criticality, and thus CityAlarm should come as an emergency alerting system that is flexible enough to adjust the detection of events. For that, an event detection paradigm had to be properly defined, assuring flexibility and scalability to the system while making it feasible.

When defining the event detection paradigm to be adopted, the literature on this subject gave some clues about the way an EI should be detected. From a common perspective, some works have detected events as a value within a numerical scale. The work in [31] detects events (through sensors’ measures) and classifies them into a numerical scale from 0 to 15. In that work, an event is always detected when its “relevancy” is greater than 0, resulting in the possibility of different events having different intensities and, therefore, priorities. In [32], social media is exploited to assess the impact of events, which may be considered when defining their priorities. The work in [33] performs an extensive survey of how risk assessment has been performed in recent years, impacting on the “evaluation” of critical events. All those works have tried to evaluate the impact of critical events, which might be associated to numerical “values” of relevance.

The biggest problem of approaches such as the one presented in [31] is that it may be too hard to assess the impact of events, especially in an urban environment composed of many concurrent and independent systems. As a consequence, it also becomes hard to provide a consistent emergency alerting system based on events associated to numerical values of intensity when there are still other variables that may also contribute to an emergency alarm, such as the concept of risk zones. For example, when saying that an event e1 has intensity 4 while an event e2 has intensity 3, we cannot say that e1 is necessarily more dangerous than e2 when generating emergency alarms for an external system since they can equally hurt people even in different proportions. Put in other words, high temperatures of $200^{\circ }$ or $300^{\circ }$ are both too dangerous for people. Therefore, if an event is assumed as critical, “less” or “more” critical may be irrelevant when generating emergency alarms.

We then define that any event of interest *e* will be associated with a sensor type *y*, fp(e)=y, with e>0 and fp(e)>0, resulting in the fact that two events e1 and e2, for e1≠e2, may have the same type, fp(e1)=fp(e2). Then, an event *e* will have d(e)=1 if it is assumed as detected, and d(e)=0 otherwise. This ON/OFF behavior indicates that two events e1 and e2, for e1≠e2, d(e1)=1, d(e2)=1 and fp(e1)=fp(e2), will be exactly the same, even if they resulted from different sensor measures. For that, what will determine whether an event is assumed as detected or not is the use of a threshold, defined by the variable $th_{\left(y\right)}$, which is configurable for each type of event in the considered city scope. For any measure taken for a particular sensor unit of a type *y* in a particular instant of time *t*, m(y,t), an event will be assumed as detected if $m\left(y,t\right)\geq th_{\left(y\right)}$ or $m\left(y,t\right)\leq th_{\left(y\right)}$, depending on the nature of the expected EI. The variable *y* indicates the type of sensed data that is associated with a group of events, and it can be freely configured according to any criteria.

Table 1 presents some examples of events of interest that can be detected in smart cities, suggesting some practical definitions for $th_{\left(y\right)}$.

While smoke (easily visible) and toxic gases (typically invisible) may sometimes be related, being detected by the same sensor unit in some cases, they are treated separately in Table 1 because they are frequently generated by different causes. For example, smoke is often generated by fire, while most toxic gases result from leakages in modern cities. Even though some toxic gases such as CO and CO${}_{2}$ may be present in typical smoke, they may also exist in the absence of fire, also resulting in potential danger to people.

Another important remark is about traffic accidents. While emergency alarms could be issued for some critical traffic accidents in smart cities, they are more related to disturbances and upsets after an accident occurs. They also do not necessarily put additional people in damage (although that may happen). In fact, parallel systems could be easily used to alert or minimize the impact of traffic accidents [34].

As a final definition, the event detection units operate checking all attached sensor units in a previously defined frequency, $fs_{\left(u\right)}$, for an EDU *u*, comparing the acquired sensed data to all defined thresholds. This is, in fact, a simple solution with acceptable computational cost and quick response when detecting critical events, when $fs_{\left(u\right)}$ is not excessively high.

### 4.2. Event Reports

After the detection of one or more events of interest, the corresponding EDU has to transmit an event report (ER) to the registered EPU. An event detection unit *u* will issue an ER composed of the ID (*y*) of all detected events, a timestamp ts of the moment it was created and the GPS coordinates of the EDU (latitude, la, and longitude, lo). The timestamp is important because it informs the EACs about the moment the events were detected (e.g., using a 64-bit UTC notation) and the proposed CityAlarm system may use some time synchronization protocol to make this information consistent [35] (although this is not a requirement). Moreover, the GPS coordinates of the EDU not only tell the location of the detected events, but also allow the EDU to be mobile, making it more effective at dynamically retrieving such coordinates from a GPS hardware module than if it just relayed information on the preconfigured location of the EDU. Hence, an event report *i* will be represented by the tuple (*u*,*i*,ts,la,lo,*E*), for i>0, with the last element (*E*) being a set containing the identification type of all detected events in time ts by the EDU *u*. Therefore, every *y* in the group of detected events must be in this set, y∈E and |E|≥1, denoting that an event report must comprise at least one detected event.

In order to make the creation and transmission of event reports more flexible, the ERs are defined in the JavaScript object notation (JSON) format [36]. The JSON is an open-standard file format that associates attributes to values, similarly to the popular extensible markup language (XML) format. As the JSON is a language-independent data format that is largely used by Internet-based applications, it may facilitate the adoption of the CityAlarm messages as input data for other systems: The emergency alarms are also generated following this format.

An event report is defined as follows, in the JSON notation. Three different EI are reported as an example (with types y1, y2 and y3), but any positive number of events can be reported, resulting in the variable $n_{\left(i\right)}=\left|E\right|$. Moreover, it is interesting to note that event reports are identified by an “ID” in the JSON message, but two different ERs can be easily differentiated by their timestamps: The use of an ID is to make the ER message more effective and formally defined. As the additional cost to transmit the variable *i* is too low, it may be worthwhile to keep this information for some tracking or registration procedures.

{   "edu": "u",   "id": "i",   "timestamp": "ts",   "gps": {    "latitude": "la",    "longitude": "lo"   },   "events": [    y1,    y2,    y3   ]}

An important issue when detecting events is the transmission frequency of event reports; two different approaches were considered when designing CityAlarm. Firstly, it could be performed in an asynchronous way every time some new event is detected. In fact, this is a simple way to implement it, but it has to deal with the possibility of an event being detected and lasting for too long. For example, when the temperature rises and triggers an Event of Heating, the temperature may remain high for hours. This long lasting occurrence of an event would not be properly reported in this asynchronous approach. From a different perspective, event reports could be transmitted in a synchronous way, according to a previously defined frequency. Doing so, if a particular event of type *y* is declared in an event report *i* but it is not in the ER i+1, we can assume that the event of type *y* is no longer detected. In fact, assuming a transmission frequency $fx_{\left(u\right)}$, for the event detection unit *u*, the periodic transmission of event reports inevitably defines a tradeoff between the use of (transmission and processing) resources and the quick alerting when detecting events. For example, for $fx_{\left(u\right)}=1s$, the use of resources may be too high when events are rarely detected but remain detected for long periods of time. On the other hand, for $fx_{\left(u\right)}=3600s$, if the same single event is still being detected, and no other event is detected by the EDU during this interval, the system will need too much time to assure that a critical condition remains active. Therefore, the value of $fx_{\left(u\right)}$ should be carefully defined, since it will be the “refresh” frequency of detected events.

In order to make the overall solution effective, the event reports need to be transmitted following a hybrid transmission paradigm, performing both synchronous and asynchronous transmissions. Such an expected transmission paradigm follows three basic rules:

1. Event reports are only transmitted to inform about at least one event of interest. If no event is detected by the EDU, which is the expected regular behavior when it is not experiencing a critical situation, no ER is transmitted;
2. Every time at least one event is detected (checked in a frequency of $fs_{\left(u\right)}$ seconds), an event report is immediately transmitted, informing about all current detected events of interest;
3. Every $fx_{\left(u\right)}$ seconds, an event report will be transmitted informing about all detected events of interest so far, but only if at least one EI is still assumed as detected. This is defined as the “refresh” transmission of detected events.

There is an important remark to be made when concerning the “duration” of an event. The adopted approach to transmit event reports is based on the premise of “positive feedback”, informing only about detected events. In such a way, an event of interest is indirectly assumed as undetected when it is not being reported. This approach may leave open the interpretation of whether an event is still being assumed as detected even when not being reported anymore. In CityAlarm, applications may decide to keep a warning state or counter-measure procedure active, even after an event changes from detected to undetected, for some period of time. This may happen because the effect of a critical event may still be felt even after it is no longer being detected, for example, in rescue operations after an event of fire [6]. Either way, the defined mechanism is flexible enough to address most concerns when alerting people to emergencies in modern cities.

**Figure 2:**
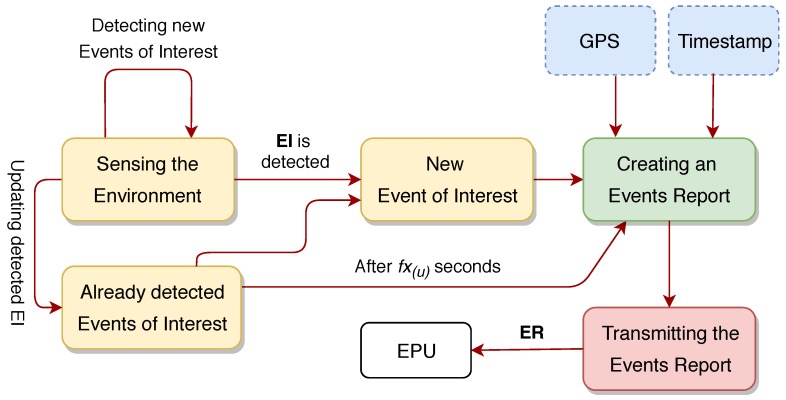
The transmission of event reports by the event detection unit (EDU).

Figure 2 presents the schematic operation of the EDU when creating and transmitting event reports.

Finally, it is worth making a remark on reliability when transmitting event reports to an emergency processing unit. As the CityAlarm system does not define a specific communication protocol or architecture, any approach could be used. However, it may be desired that some reliability service is provided, for example employing the transmission control protocol (TCP) of the standard Internet protocol stack. Moreover, such transmissions could also be performed exploiting some security mechanism, for example, encrypting the transmitted ER.

## 5. Generating Emergency Alarms

After detecting events, an EDU must transmit event reports to the registered emergency processing unit, exploiting a networking approach. In the EPU side, all ER messages are received, processed and aimed at the generation of emergency alarms. The following definitions describe how such alarms are generated and delivered to external systems.

### 5.1. Defining an EA

An EPU continuously receives event reports from one or more EDU, generating emergency alarms according to the following definitions:

- Every event report results in the generation of a single emergency alarm.
- Every emergency alarm is associated with a unique event report.
- All emergency alarms are independent. Multiple messages of the same EA may be transmitted to any number of emergency alarm clients, including to no EAC at all.

When dealing with risk management and recovery from hazards in the context of smart cities, there are four important pieces of information that must be considered when processing a disaster [33]: (a) Its magnitude, (b) its related geographical scope, (c) its temporal significance and (d) its impact duration. While such pieces of information could be considered as components of a disaster, the CityAlarm is not a disaster management system itself, but an integrated emergency alerting system. In this sense, an emergency alarm is primarily related to the elements that may cause a disaster, and not the consequences or their duration. Moreover, as an alerting system, the magnitude of an emergency is the most relevant piece of information to be computed and delivered, but CityAlarm also aggregates other relevant information (notably the geographical scope and the temporal significance) to compute the magnitude level of each alarm, assuring a more comprehensive perception of emergencies for the requesting applications.

As a fundamental assumption of the proposed approach, the magnitude of an emergency is directly related to the number of detected events. As critical events are processed as logical (ON/OFF) variables in CityAlarm, it is not the actual sensed data that is relevant when alerting people to an emergency, but the capacity of the system to detect multiple variables that are assumed as being in a critical state. Once again, the proposed system is a solution for emergency alerting and thus the simultaneous detection of multiple critical events is more relevant than the detection of a single critical event with very “dangerous” detected data. Nevertheless, as mentioned before, other variables will also impact the computing of the magnitude of an emergency alarm, referred to herein as its “severity level”.

An emergency alarm *a* will be represented in the JSON format in the same way as in an event report. An emergency alarm will contain most of the information that is already in the corresponding ER, plus two additional pieces of information: The ID of the alarm, *a*, and the severity level of the EA, defined as $sl_{\left(a\right)}$. An example of an emergency alarm is presented as follows, for three reported events of interest in a received ER:

{   "id": "a",   "severity" "sl",   "timestamp": "ts",   "gps": {    "latitude": "la",    "longitude": "lo"   },   "events": [    y1,    y2,    y3   ]}

For the emergency alarms, the identification of the EDU (*u*) was suppressed since it may be considered as an internal parameter that can be processed by the EPU for some additional procedure, but that is irrelevant for an EAC.

### 5.2. Risk Zones

Besides the number of detected events of interest, the severity level of an EA will also be impacted by the geographical scope and the temporal significance of the detected events. The geographical scope is modelled by CityAlarm as risk zones, while the temporal significance is directly considered when computing the emergency magnitude.

A risk zone *z*, for z>0, is defined as a circumference with center ($x_{\left(z\right)}$, $y_{\left(z\right)}$) and radius $d_{\left(z\right)}$. All events detected inside that circumference will be under the influence of the corresponding risk zone, which means that an EDU must always be inside at least one risk zone. Every risk zone will also have an impact level, $r_{\left(z\right)}$, which is a positive integer number defined according to the particularities of the considered city. The values of $r_{\left(z\right)}$ will range from 0 (lowest level of risk) to rmax (the highest level of risk), and 0<rmax, assuring the definition of at least two different types of risk zones. Additionally, it is also possible that different risk zones have the same value of $r_{\left(z\right)}$.

As additional definitions, the risk zones may overlap, eventually resulting in an EDU being inside two or more risk zones. In this case, it is assumed that the EDU is effectively inside the risk zone with the highest value of $r_{\left(z\right)}$. Another relevant remark is that if there is no definition for a risk zone comprising a particular EDU, that EDU is considered to be in a risk zone with the lowest level of risk (assumed as 0).

### 5.3. Computing the Severity Level

An emergency alarm has the objective of telling what the nature of an emergency is (through the number and types of the detected events), where an emergency is occurring (through its GPS coordinates), when it happened (through a timestamp) and what its impact is (through the computed severity level, $sl_{\left(a\right)}$). Since the first three variables are already known, the challenge is how to compute the severity level of an emergency.

In order to make the CityAlarm a flexible but still effective system for emergency alerting, the severity level associated to each emergency alarm is computed considering three different components: A) The number of detected events in a received event report, b) the relative impact of the risk zone associated to the ER, and c) the temporal significance of the ER measured as a combination of date and time information. All these three variables are completely independent parameters (events may happen anywhere and anytime) and thus they will be processed independently, resulting in a numerical perception of the severity impact of each emergency alarm.

As already mentioned, all events of interest are equal when alerting people to a critical situation. Hence, the number of reported (detected) EI in the event report *i*, $n_{\left(i\right)}$, is the relevant piece of information when computing $sl_{\left(a\right)}$, and not the types of the detected events. In this context, we define that the number of events of interest to be considered when computing $sl_{\left(a\right)}$ is limited to 5, creating the same scope of significance for the entire system when assuming any possible configuration of the EDUs. In fact, an emergency situation with 5 concurrent events of interest being detected at the same location is already extremely severe and such a high level of severity would be almost the same if more EI were detected, in practical means. Moreover, even if this constraint did not apply, an ER with more than 5 events would be rare in an urban scenario [37,38]. Thus, this decision is intended to make the proposed system more practical, creating a consistent and unified scope of significance for the emergency alarms. As an important remark, when more than 5 EI are being detected, the EDU may choose which EI will be reported, this being basically an implementation issue.

Considering the risk zones, the challenging issue is to define the actual risk of each zone; a challenge that could consider factors such as the amount of people living in the area and the presence of potentially dangerous infrastructure (such as a gas station nearby, an electrical substation or a factory). As the minimum acceptable value for the impact level of any risk zone is 0, the designer of a CityAlarm-based application only have to pick a valid rmax>0 and choose a value for every $r_{\left(z\right)}$ in this range.

Finally, the most tricky variable is the temporal significance of an emergency alarm. In order to make it tractable, the temporal significance is modelled as $t_{\left(a\right)}$, a numeric value ranging from 0 to tmax. While it may resemble the way risk zones are processed, since $t_{\left(a\right)}$ is also within the range of acceptable numerical values, $r_{\left(z\right)}$ and $t_{\left(a\right)}$ have completely different scopes. While a risk zone associates a risk factor to the area where an emergency alarm is coming from, the value of $t_{\left(a\right)}$ indirectly indicates the success likelihood of emergency mitigation and rescue operations according to the day of the week and the hour of the day. For example, if an event happens during work hours on weekdays, its impact might be softened because emergencies could be more quickly “solved” (or the opposite, depending on the specifications of the system and the considered urban area to be monitored). However, an emergency could be boosted during dawn on weekends, when emergencies may take longer to be addressed and even potential victims may be asleep.

The value of $sl_{\left(a\right)}$ is computed as expressed in Equation Equation (1), which is the adopted emergency magnitude equation.

(1) $$sl_{\left(a\right)}=\left(n_{\left(i\right)}\times 20\times f_{e}\right)+\left(\frac{r_{\left(z\right)}\times 100}{rmax}\times f_{r}\right)+\left(\frac{t_{\left(a\right)}\times 100}{tmax}\times c_{\left(t\right)}\times f_{t}\right)$$

The formulation presented in Equation (1) is normalized, resulting in a value for the severity level in the range $0\leq sl_{\left(a\right)}\leq 100$. The constants $f_{e}$, $f_{r}$ and $f_{t}$ are used to tune the variables employed when computing $sl_{\left(a\right)}$ since this may be desired by a particular application. For example, if the number of detected events is twice as important as the definitions of risk zones, we might have $f_{e}=0.6$ and $f_{r}=0.3$. Nevertheless, it is expected that $f_{e}+f_{r}+f_{t}=1.0$ and a “regular” implementation would define $f_{e}=f_{r}=f_{t}=1.0/3$.

Another variable that deserves proper explanation is $c_{\left(t\right)}$. In fact, the definition of $t_{\left(a\right)}$ is highly subjective: For example, for tmax=100, an application can define that $t_{\left(a\right)}=100$ for weekdays (from Monday to Friday), $t_{\left(a\right)}=70$ for Saturdays and $t_{\left(a\right)}=50$ for Sundays. However, the value of $t_{\left(a\right)}$ may change throughout the day. For that, the variable $c_{\left(t\right)}$ could be obtained from any function in time *t*, ranging from 0 (when the influence of $t_{\left(a\right)}$ is cancelled) to 1.0.

The definition for $c_{\left(t\right)}$ may be obtained from some configurable function. As a standard reference for CityAlarm, we propose a Gaussian distribution [39], altering the value of $c_{\left(t\right)}$ along the considered day. The employed Gaussian distribution is presented in Equation (2).

(2) f(x;σ,μ)=e−x−μ2−x−μ22σ22σ2

**Figure 3:**
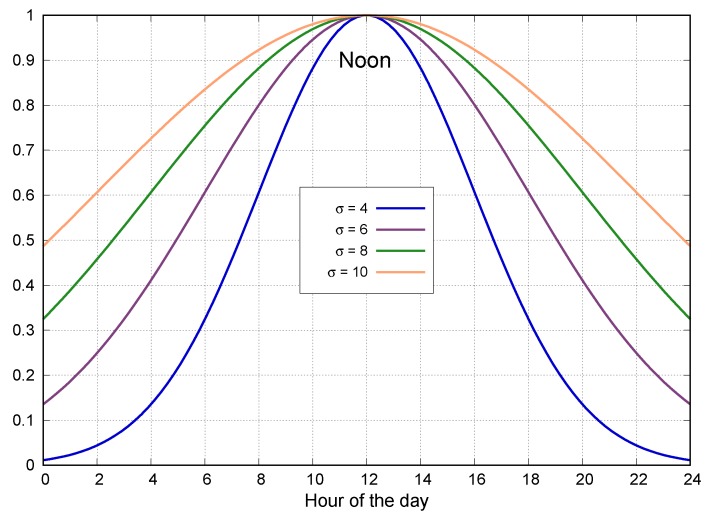
Four different Gaussian distributions when computing $c_{\left(t\right)}$.

If the hour of the day is measured from 0 to 24, the value of μ=12 would determine the highest value for the Gaussian function at noon. Then, the value of σ (standard deviation) would define how the Gaussian function would “behave” according to the hour of the day. Figure 3 presents four different distributions for different values of σ. It is worth noting the different values of f(x;σ,μ) from 6 AM to 6 PM, which are the hours when most people are more active.

The interpretation for the function described in Equation (2) depends on the characteristics of the considered system. If an emergency has a lower negative impact around noon, then $c_{\left(t\right)}=1-f\left(x;\sigma ,\mu \right)$. If the opposite applies, then $c_{\left(t\right)}=f\left(x;\sigma ,\mu \right)$. In both cases, the value of $c_{\left(t\right)}$ may even be 0, suppressing the impact of the temporal significance for the computation of $sl_{\left(a\right)}$.

### 5.4. Transmitting Emergency Alarms

The ultimate objective of the proposed CityAlarm system is to deliver emergency alarms to any number of clients (emergency alarm clients), who can exploit those messages in different ways. To achieve that, after generating an EA, some efficient mechanism has to be employed by the EPU to send emergency alarms to every EAC that is intended to receive them, and there are many possible ways to do that [40].

In order to provide a flexible, efficient and already validated mechanism to deliver emergency alarms, we propose the use of the Message Queuing Telemetry Transport (MQTT) protocol as the basis for EA dissemination in CityAlarm. The MQTT [41,42] is an application-layer protocol based on the paradigm of publish-and-subscribe message exchange. Operating over the TCP/IP protocol stack (and exploiting the reliability of TCP), the MQTT operates around the concept of “broker”: An element subscribes to a broker to receive notifications, while another element publishes messages directly to the broker, indirectly notifying the original requesting element. In this paradigm, the publishers and the subscriber units do not interact directly, making the broker responsible for handling the receiving of messages and the insertion of new subscribers to the system. These particular characteristics have made the MQTT a very popular protocol for IoT and smart city applications, with a lot of practical implementations [43].

**Figure 4:**
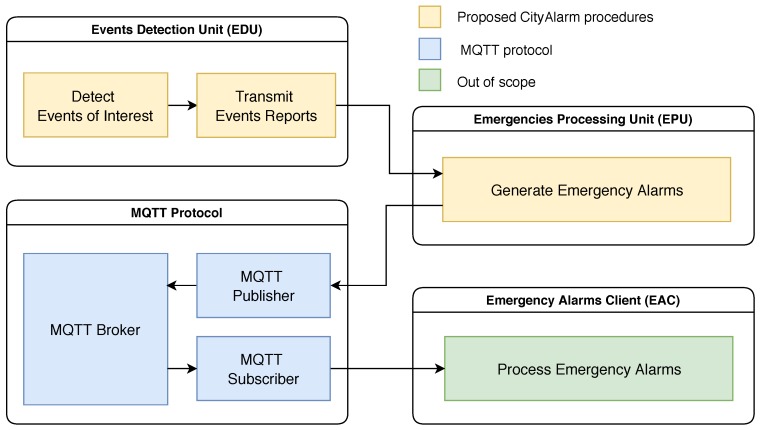
The communication flow in the proposed system, assuming the use of the Message Queuing Telemetry Transport (MQTT) protocol.

As initially depicted in Figure 1, the EACs are expected to subscribe to some EPU to receive emergency alarms, in a process simply referred as “EAC subscription”. When the MQTT is employed to handle the transmissions of EA messages, the communication scope of CityAlarm is expanded, resulting in the architecture presented in Figure 4. The presented communication flow must be considered along with the definitions in Figure 2, which displays the frequency of transmissions of event reports from any EDU to an EPU. Putting all this together, events of interest can be detected and emergency alarms can be generated and transmitted for any kind of processing, making CityAlarm a flexible and scalable solution.

It is interesting to note that the use of MQTT does not interfere in the core operations of CityAlarm, which are the detection of events of interest and the generation of emergency alarms, and thus that protocol could be replaced by another communication solution without compromising the proposed emergency alerting system. Nevertheless, due to the widespread use of MQTT in IoT-based applications, the implementation of the EPU and the EAC can benefit from its flexibility and effectiveness, easing the practical use of CityAlarm.

## 6. Tests and Experiments

The proposed emergency alerting system has been comprehensively specified in previous sections, which described the characteristics and operation of the elements related to the detection of events and reporting of emergency alarms in urban areas. However, due to its particularities, it may be worthless to perform comparisons with other systems, especially because CityAlarm is a flexible multi-events distributed emergency alerting system with some innovative ideas, such as the way events of interest are detected and also the separation of the concepts of events and emergencies. On the other hand, solutions in the literature have treated this problem on a reduced scale, usually addressing only some particularity of emergencies in modern cities. Therefore, the validation of the proposed system was centered on practical implementations of CityAlarm in real applications, attesting that it is ready to be exploited in urban scenarios.

### 6.1. A Proof-of-Concept of CityAlarm

In order to provide a proof-of-concept to support the validation and practical use of the proposed approach, all three units of CityAlarm were implemented. The implementations follow the definitions in this article and they are freely and openly available in a public repository at https://github.com/lablara/cityalarm.git. The codes (written in the Python3 programming language) and hardware specifications are valuable resources when attesting the effectiveness of the proposed approach at providing the expected services. Moreover, they are also a reference when employing the CityAlarm system in practical applications: All the codes can be easily modified to attend to the characteristics of any smart city scenario.

An event detection unit is expected to sense the environment, to detect events of interest and to transmit event reports to a registered EPU (directly configured in the code or informed as a command-line argument). This means that the EDU must be able to interact with a group of different sensor devices. For that purpose, the EDU was implemented using the Raspberry Pi platform (https://www.raspberrypi.org), which is a very popular open-source system-on-a-chip (SoC) platform that has been largely used to create sensor-based networks, IoT nodes and smart city applications [13,21]. However, an EDU could be easily implemented on any hardware platform, since the expected services are still provided.

An EDU may detect one or more type of event, according to the number of sensor devices employed to construct the unit. The users are encouraged to construct their own event detection units according to the particularities of the target scenario. In order to employ an easy way to attach various different sensors to the same EDU, we exploited the GrovePi+ hardware framework (http://wiki.seeedstudio.com/GrovePi_Plus/), facilitating the insertion of multiple heterogeneous sensors to a single Raspberry Pi board. Besides creating a standard interface for hardware interaction, following a plug-and-play paradigm, the GrovePi+ enables the communication of multiple analog and digital sensor devices to the same Raspberry Pi board in a solderless and intuitive way without directly using A/D converters. In practical means, this suggested that hardware modules make the implementation of the EDU simpler and easier, concerning both the hardware connections and the software programming, since users might be more concerned with the details of the CityAlarm system than in hardware wiring and sensor interfacing.

Table 2 summarizes the adopted hardware components used to construct an EDU in this proof-of-concept. The number and type of the employed sensor device can be easily changed depending on the available components and the characteristics of the target scenario.

There are some interesting remarks to make about the employed sensor modules. First, the same sensor device that is used to retrieve relative humidity could also be used to retrieve temperature data. However, the temperature range measured by the “DHT11” sensor is too short (from $0^{\circ }$ to $50^{\circ }$) and thus we decided to use an additional device to retrieve temperature (from $-40^{\circ }$ to $125^{\circ }$). Concerning the employed air quality sensor device, it was designed for comprehensive monitoring over indoor air conditioning, being able to detect a wide scope of harmful gases (carbon monoxide, alcohol, acetone and formaldehyde, among others). However, it is basically a qualitative sensor, not a quantitative one. Nevertheless, as an EDU may not measure the precise concentration of a specific gas but a dangerous concentration of gases, that sensor is well suitable for event detection.

**Figure 5:**
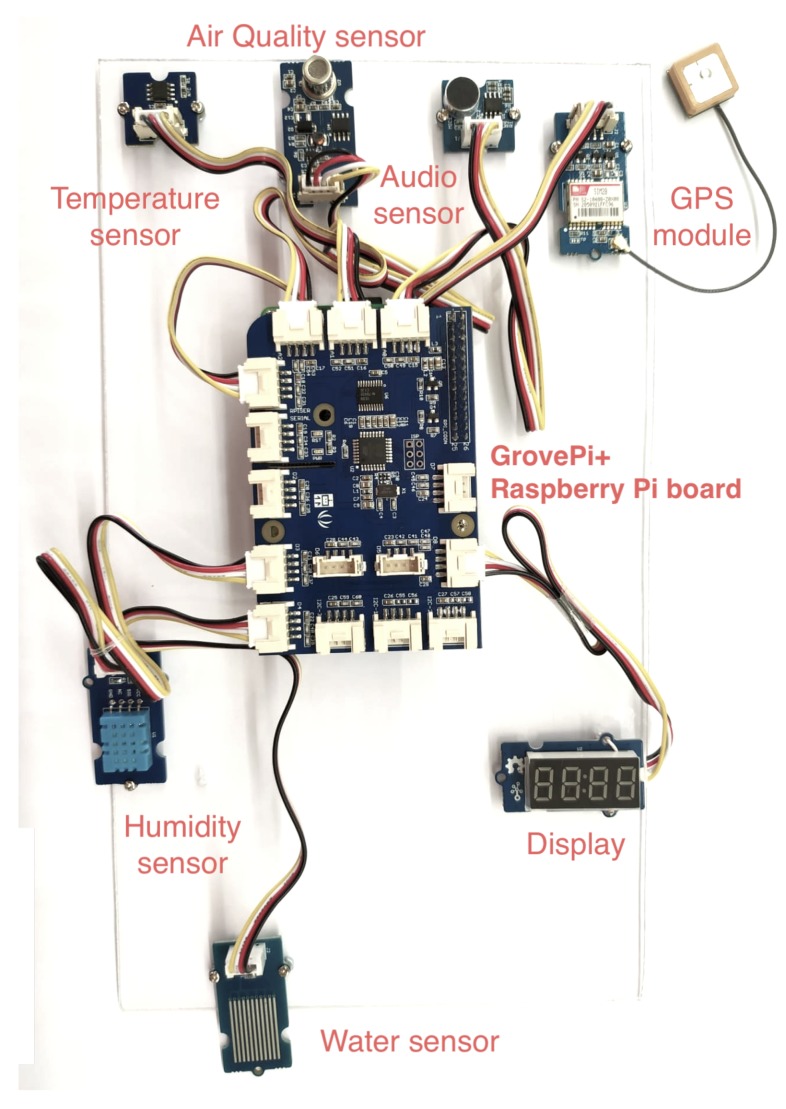
An EDU created with Raspberry Pi and GrovePi+. All employed components in this example are compatible with the Grove system.

Figure 5 presents the EDU created for the proof-of-concept, according to the definitions in Table 2. All the components were attached to an acrylic plate to facilitate the execution of the experiments.

When deploying an EDU such as the one depicted in Figure 5, one should note that the sensor devices may need to be calibrated according to the information provided by the manufacturer. Besides that, users should also pay attention to the use of the GPS module, which can be compromised if the GPS signal is not properly acquired (for example in some indoor environments). However, in cases where the GPS fails to retrieve the current position, users can define a default GPS configuration for the EDU.

The reference implementation of the EDU was configured with the following default parameters: u=1 (if more than one EDU is employed, each one must have a different ID), $fs_{\left(u\right)}=5s$ and $fx_{\left(u\right)}=60s$, making the ER refreshing frequency 12 times higher than the sensing frequency. The events of interest that can be detected in this particular EDU implementation were configured according to the sensor devices described in Table 2 and the definitions in Table 1. In this case, the threshold for each event of interest was settled to parameters that can be easily reproduced in a indoor Laboratory. Considering the five different sensor devices defined in Figure 5, this reference implementation allows the identification of up to six different events of interest, since the temperature sensor can be used to trigger both an event of heating and an event of freezing (but obviously not at the same time). Either way, users can add any type of sensor device that can provide data related to some emergency situation.

Finally, the IP address and TCP port of the EPU has to be known by the EDU. Both information can be provided as command-line arguments, or be configured directly in the code. As a default parameter, the TCP port of the EPU was defined as 55055: An EPU continuously waits for connection requests at this port, processing new connections in separated threads to receive the event reports in the JSON format.

The EPU is a unit with only software implementation and thus it can be executed in any device (including in an online regular computer). Users must be concerned only with the definitions of the parameters specified in the proposed CityAlarm system. So, in this reference implementation, the considered default parameters are $f_{e}=0.4$, $f_{r}=0.3$, $f_{r}=0.3$, μ=12 and σ=6 (as in Figure 3), resulting in a configuration that slightly favors the number of detected events (higher value of $f_{e}$) and the magnitude of the emergencies is higher when closer to noon (defined Gaussian function). Moreover, for the value of $t_{\left(a\right)}$ in equation Equation (1), it was defined that $t_{\left(a\right)}=tmax$ from Monday to Friday, $t_{\left(a\right)}=2*tmax/3$ on Saturdays and $t_{\left(a\right)}=tmax/3$ on Sundays. Finally, rmax=100 and tmax=100.

The second relevant configuration to worry about in the EPU is the definition of risk zones. They have to be defined considering GPS coordinates and a radius in km (this basic implementation takes areas in the city of Porto, Portugal, for reference purposes). The correct value of $r_{\left(z\right)}$ in Equation (1) is then computed considering the Haversine equation [44].

Finally, the EAC is implemented as a simple client that receives emergency alarms in the JSON format and prints them on the screen, showing that EA messages can be correctly received.

The EPU and the EAC communicate through the MQTT protocol. As a reference, the chosen MQTT Broker is the Mosquitto (https://mosquitto.org), an open-source and popular MQTT Broker that has supported many IoT and Smart City projects. In the conducted experiments, the Mosquitto Broker was executed on a dedicated Raspberry Pi 3B+ board and the topic registered in the Mosquitto broker was “CityAlarm_EPU1” since it is published by the EPU with u=1. Additionally, concerning the CityAlarm’s units, the MQTT messages are handled exploiting the paho-mqtt Python library (https://pypi.org/project/paho-mqtt/).

Putting together all these units, the CityAlarm performed as expected. Events of interest are detected and declared in event reports (transmitted from the EDU to the EPU). The EPU then receives the ER and generates emergency alarms, which are published in the MQTT Broker. The EAC subscribes to receive emergency alarms, which are promptly received when published by the EPU. All three units were implemented with a “debug” option to print trace massages on the screen, presenting the operation steps of each unit.

As a final remark, the corresponding codes of this reference implementation are organized into three distinct directories: EDU, EPU and EAC. The EDU contains the files edu.py (main code of the EDU), elementsEDU.py and moduleGPS.py; the EPU contains epu.py (main code of the EPU), elementsEPU.py and eaTransmitter.py; and the EAC has the simpleEAC.py file.

### 6.2. An EAC to Plot Emergencies on a Map

The presented proof-of-concept is an important reference for implementations following the definitions and procedures of CityAlarm. With the provided codes, more complex applications can also be developed, supporting effective services in urban areas. As an example of this potentiality, we created a more complete EAC to receive emergency alarms and to plot pins on a map indicating where emergencies are happening. Additionally, the severity level and type of event that generated the EA are also presented. As discussed in Section 2, the action most usually performed during an emergency is the issue of warning messages, as performed by this implemented EAC.

When emergency alarms are received, this EAC inserts them into a list of EA objects, which are all processed to be displayed on a map according to the GPS coordinates presented in the alarms. When new alarms from the same GPS position are received, the list is updated, replacing the older alarm by the newest received EA. The idea is to always display the most recent alarms on the map. Moreover, as an EDU may cease to transmit event reports when no EI is being detected, a thread in the EAC checks all EA in the list, removing old alarms (the default verification frequency was settled in 120 s). This creates an effective and robust mechanism to visually alert people to current emergency situations.

In order to support the use of maps, the folium Python library (https://pypi.org/project/folium/) was employed. That library facilitates the use of the Leaflet JavaScript library (https://leafletjs.com), which is an efficient and very popular library to interact with different functions related to maps. Using the folium library, the implemented EAC constantly generates an “.html” file containing the desired map, which can be directly accessed or easily embedded into any web-based applications.

**Figure 6:**
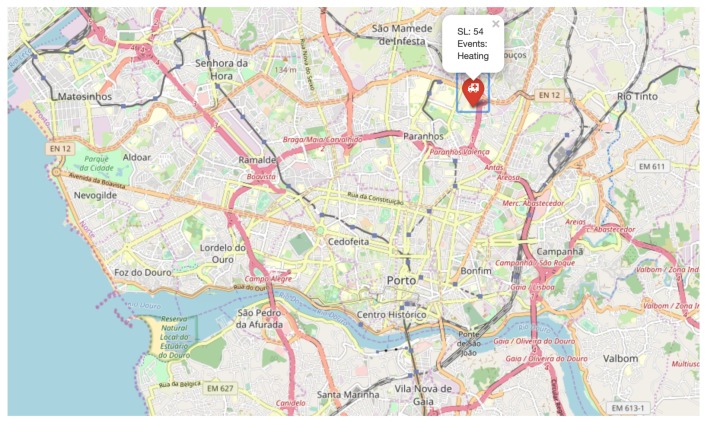
The city of Porto with one emergency alarm being reported.

Figure 6 presents a map of the city of Porto, Portugal, with one EDU reporting an emergency.

**Figure 7:**
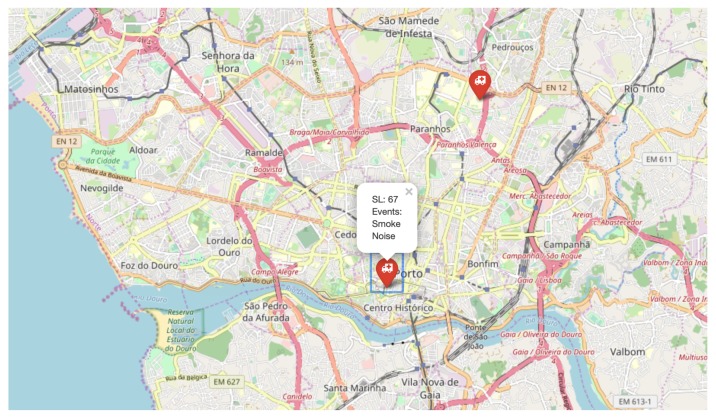
The city of Porto with two emergency alarms being reported.

In order to test the impact of different risk zones, the implemented EAC was also executed considering more than one EDU in operation, positioned in different zones. In this scenario, three different risk zones were defined, considering the city of Porto. For two EDUs in different zones, a result of the EAC is presented in Figure 7.

In Figure 7, two EDUs are detecting events at the same time, but at different locations. As the same EPU is contacted by both EDUs, and that EPU is defining the same temporal significance for the alarms, the difference between the alarms is the risk zone. In this case, the alarm generated from measures at the Faculty of Engineering of the University of Porto (with ID 1) had $sl_{\left(1\right)}=54$, while the alarm from the Rectory of the University of Porto (with ID 2; depicted in Figure 7) had $sl_{\left(2\right)}=67$.

**Figure 8:**
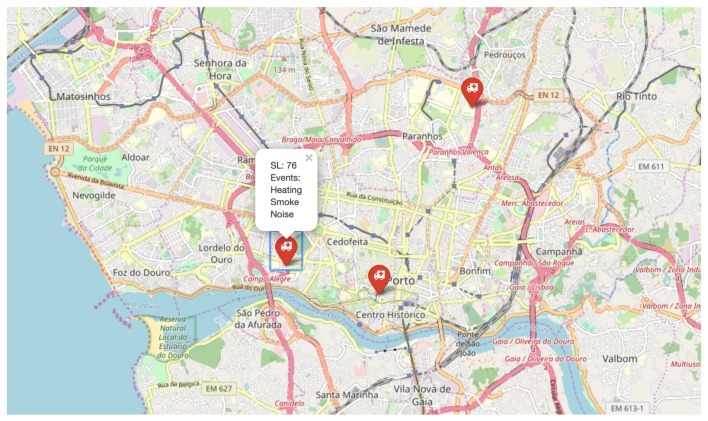
The city of Porto with three emergency alarms being reported.

Finally, a third EDU reported an emergency alarm comprising three different events, as depicted in Figure 8. The computed severity of that alarm was $sl_{\left(3\right)}=76$.

The developed EAC is a practical application of CityAlarm that can be exploited immediately, taking benefit of the services of the proposed system. This EAC implementation is also available in the same public repository, located at https://github.com/lablara/cityalarm.git, in the directory “/EAC_MAP”.

### 6.3. Implementation and Deployment Issues of CityAlarm

When implementing and deploying the elements of the CityAlarm system, some relevant issues may arise demanding special attention. Some of those issues were experienced during the development of this approach, while other issues are expected to be relevant when large CityAlarm-based applications are deployed. In both cases, knowing them may be valuable for practical use of the proposed approach.

The CityAlarm system relies on a number of event detection units that are scattered over an area to be monitored, covering part or the entire area of a city or metropolitan region. In this sense, the deployed EDUs must be connected to some network. In practical terms, such connections may be in the form or wired or wireless links and any technology could be employed (e.g., Wi-Fi, Wimax, 5G, LoRaWAN, Ethernet), since the target EPUs can be accessed. While the proposed approach does not define a specific communication technology, the Internet protocol stack is the modern “standard” for inter-networking and the CityAlarm’s units will operate over TCP/IP or UDP/IP communication flows. However, while the Internet protocols are de facto solutions for interconnections, Internet-based communications have a lot of issues that may severely impact the operation of CityAlarm.

The delivery of event reports from an EDU to an EPU and transmissions of emergency alarms from an EPU to an EAC should be performed as fast as possible, reducing the transmission delay. However, there are no delivery guarantees in Internet-based connections concerning transmission latency, which may be too severe for emergency detection and alerting. Moreover, in order to assure some level of reliability, the CityAlarm’s units should communicate through TCP connections, which inevitably adds latency to the communications when retransmissions are performed. The end-to-end latency may be hard to predict and reduce, but a common approach to reduce the average delay is employing links with higher bandwidth and lower transmission demands.

Another relevant issue is effectiveness. The transmission link of an EDU may fail, disconnecting event detection in some area. While this may be hard to avoid in most cases, some critical areas may employ EDUs with redundant links: A cheaper/faster link could be used as the default transmission option, which is replaced by a secondary more expensive backup link (for example a 4G connection) when a failure is detected in the main link.

While it was not a major concern when designing CityAlarm, some security issues may be relevant for consideration for some applications. However, there will be different concerns about security, each one demanding proper solutions. In some cases, event detection may create concerns about confidentiality and authenticity, demanding some level of authentication. Additionally, in order to avoid false alarms, integrity may also be desired. For all these concerns, cryptography is the most effective tool to protect data and authenticate the elements of CityAlarm, at the cost of additional processing load. Nevertheless, the CityAlarm’s units may still be vulnerable to denial of service (DoS) attacks, compromising the availability of the system. The CityAlarm system will be as protected against DoS attacks as any other Internet-based system, which implies that constant monitoring for attacks and fast response to them may be required in some cases.

Besides networking issues, there are some project and deployment issues that should also be carefully considered. The first of them is the number of EDUs that need to be employed. An EDU will detect events in its deployment area and thus some events may be undetected depending on the configuration of the region (e.g., number and characteristics of the buildings) and the number of employed EDUs. In a simple analysis, a greater number of deployed EDUs will increase the granularity of the system, also increasing the capability of the system to detect events of interest. However, the available EDUs to be deployed will depend on the budget of the emergency detection system. A second issue concerning monitoring is the nature of the sensing units. Sensors may be designed to perform indoor or outdoor monitoring and an EDU should be assembled taking more suitable sensors to its expected operation. However, such a decision will not only affect the operation and reliability of the event detection procedures, but also the final cost of an EDU. As a result, it is expected that a CityAlarm-based application will employ different EDUs in a city, with different costs and deployment concerns.

As a final concern, the units of the system should be designed to reduce their overall energy consumption. The adoption of the Raspberry Pi platform for the construction of the proof-of-concept was also made based on this principle, keeping energy consumption low. However, as the units may be created employing any hardware, this concern should still be considered when designing them.

## 7. A Smart City Perspective of Emergency Alerting

The CityAlarm is intended to detect any kind of measurable critical event, processing them as emergency alarms that have spatial and temporal significance. Besides providing a feasible way to track and visualize emergency situations in urban areas, the proposed system also provides valuable information for many smart city applications, which may exploit CityAlarm as a concurrent supportive system. This may create many promising possibilities for smart city scenarios.

Several smart city applications have been designed around the major concern of hazards. In this scope of problem, prediction, rescuing and alerting procedures are some of the services that have been supported by sensor network initiatives. In this sense, many solutions have been proposed, addressing different challenges and particularities of critical situations in smart cities [45,46,47,48]. While promising, however, much of such solutions could be enhanced by exploiting the services provided by CityAlarm.

Event detection has been a challenging task for smart cities, mostly due to the great number of different events that may be detected from different sensing units [8]. In [6], events are detected exploiting social media data, making people a particular type of sensor. The proposed approach in that work can detect a lot of events, but its response to critical events could take too long, which may not be acceptable for quick emergency alerting. Nevertheless, if the work in [6] could be combined with CityAlarm, quicker responses to emergency situations could be achieved, while obtaining more of the details of the detected events through data mining. In [49], smartphones are used as sensing units for emergency detection, which are inherently mobile. Once again, CityAlarm could provide additional data to enhance the performance of that system, complementing the crowd-sensed data. In another example, authors in [50] proposed a management and alerting system for earthquakes. In fact, we can expect that earthquakes can also indirectly cause fire and gas leakages, which should also be monitored and alerted. If the system proposed in [50] was integrated with CityAlarm, a more complete perception of earthquake hazards could be achieved.

Another promising research field is sensor prioritization exploiting the detection of emergencies. When considering the deployment of sensor networks in smart city scenarios, some sensors may be assumed as being more relevant than others, and such different relevance levels (priorities) can be used to enhance the performance of a network while reducing the overall expenditure of resources such as energy and available bandwidth [12]. For that purpose, there are many ways to compute and exploit such priorities. In [51], a fuzzy-based decision mechanism is used to compute priorities according to characteristics of the environment and the sensing and processing resources of sensor nodes, resulting in a uniform scope of prioritization for the entire network. Likewise exploiting prioritization, the work in [52] dynamically changes the way packets are routed according to their relevance for the application. From a wider perspective, the work in [53] differentiates sensor nodes in a smart home scope, which may be beneficial when quickly delivering sensitive data. In all those cases, the CityAlarm system could be helpful when differentiating the most relevant sensors and consequently the most relevant data, taking advantage of the concept of emergencies and their associated severity levels.

In general, any application that requires some “differentiation” could benefit from the adoption of CityAlarm as a parallel supportive system. A sensor node is usually designed to collect one or more type of information, which is delivered to some node or network for further processing [2,54]. In this context, many applications have been designed based on different strategies when leveraging information, trying to achieve adequate performance for the expected services of the nodes, on an urban scale. In this sense, we believe that the proposed system is not only a valuable resource for efficient emergency alerting, but it is also an effective supportive system for sensor-based event-centered services in smart cities.

An additional relevant remark about the practical use of CityAlarm is its implementation. This article provided an affordable implementation of the three elements of this approach (EDU, EPU and EAC), employing a popular hardware platform. However, there are different ways to achieve the same results or even to provide a more effective implementation for spread use in large cities. For example, dedicated components can be designed and printed, creating units that are specialized for the particularities of emergency detection of any city. From a similar perspective, some development frameworks and programming tools could be used to enhance the creation of the units, such as the Node-RED (https://nodered.org), which facilities hardware programming. As CityAlarm is a flexible solution that does not depend on a particular hardware, such development approaches could be beneficial for some applications.

Finally, concerning the scope of smart cities, with a huge amount of data being produced continuously, the proposed CityAlam system could be extended to exploit additional techniques, such as edge computing. When bringing computation and data storage closer to where events are being detected and processed, the EDU could be divided in multiple processing cells for faster detection of some more critical events such as noxious gases (depending on wind conditions, they may be hard to quickly identify in some areas) and fire. Concerning the IoT scope of problems, some works have exploited edge computing to achieve some kind of efficient and quick event detection, as in [55,56]. Additionally, frameworks and models on how to exploit edge computing in IoT systems for some specialized detection have also been designed. In [57], the authors proposed a complete approach to create complex IoT systems based on edge computing, potentially providing support when developing applications in smart city scenarios or extending systems like CityAlam. This concept of “edge of things” further extends the usual paradigm of IoT, with direct application on event detection and alert. In a different approach, the work in [58] also exploited edge computing for efficient distributed processing of sensed variables.

## 8. Conclusions

Emergency management in urban areas was a major concern even before the Computing Age, with warning and counter-measure mechanisms being developed to reduce casualties and economic losses. Many cities in human history have been devastated by incidents like earthquakes, volcanic eruptions, fire and flooding, which have demanded the development of different and sometimes error-prone detection and alert mechanisms. Recently, with the advent of sensor-based monitoring technologies, new possibilities for emergency management and alerting have been created, making those processes faster and more reliable. In this still evolving scenario, CityAlarm comes as an effective and flexible system for distributed emergency detection and alerting for different types of applications.

Relying on the flexibility of distributed sensors to retrieve information about a variety of environmental variables, the proposed approach defines different tiers for emergency processing, facilitating its adoption in large cities as long as it assures scalability to the system. Moreover, the separation of “events” and “emergencies” as different but related concepts creates a strong perception of emergency situations and the corresponding alerting process. As a consequence, CityAlarm can be easily adapted and configured to the particularities of any city, making this approach a very suitable candidate for handling emergency detection and alerting in modern urban areas. In this sense, although CityAlarm might be theoretically employed in any risk-prone environment, the modern cities were chosen as the target scenario for the proposed system mostly due to their inherent characteristics. Nevertheless, any scenario that can benefit from distributed event detection could also exploit CityAlarm, as in military surveillance, weather monitoring and industrial automation.

The procedures, variables and communication flows specified in this article clearly define how critical events can be detected and how emergency alarms can be generated and delivered. While the presented definitions can already be used to guide the practical implementation of CityAlarm, this article also demonstrated the fully-operational implementation of the system, for all the proposed units: EDU, EPU and EAC. That implementation, which is openly available at the GitHub repository https://github.com/lablara/cityalarm.git, allows fast development of real applications following the proposed approach, not only boosting its practical exploitation but also supporting further validations of this approach. Hence, since the proposed system was properly defined and its implementation is public and open, many different CityAlarm-based applications can be envisioned and developed, enhancing its practical contributions.

As future work, additional experiments will be performed to create a database with emergency information from a group of selected cities. Such a database, composed of received emergency alarms during a certain period of time, can provide valuable information about critical incidents in those cities, which could be exploited for different functions. For example, neighborhoods with a historically higher number of alarms (or alarms with higher severity) can be identified, potentially helping the government when planning action. That data can even be combined with other databases, such as dangerous weather conditions or energy blackouts, providing a very comprehensive perception of emergencies in the considered cities.

## Figures and Tables

**Table 1:** Some examples of typical events of interest in smart cities.

Event Description	Scalar Data	Type (*y*)	Threshold ($th_{\left(y\right)}$)
Heating	Temperature	1	m(1,t)≥60 °C
Freezing	Temperature	2	m(2,t)≤−20 °C
Low Humidity	Relative humidity	3	m(3,t)≤10%
Smoke	C0${}_{2}$ concentration	4	m(4,t)≥5000 ppm
Toxic Gases (leakage)	Ammonia (NH${}_{3}$)	5	m(5,t)≥35 ppm
Heavy Rain	Rain precipitation	6	m(6,t)≥10 mm/h
Earthquake	Seismic magnitude	7	m(7,t)≥6.5 M${}_{L}$
Noise Pollution	Audio signal strength	8	m(8,t)≥100 dB
Dangerous Radiation	Radiation	9	m(9,t)≥200 rad
Blast Wave	Air pressure (or wind speed)	10	m(10,t)≥5 psi
Strong Wind	Wind speed	11	m(11,t)≥80 km/h
Low Luminosity	Light intensity	12	m(12,t)≤1 Lux
Heavy Snowing	Snowfall	13	m(13,t)≥50 cm/day
Dangerous Dam Level	Dam water level	14	m(14,t)≥95%

**Table 2:** Employed hardware components in this implemented EDU.

Component	Model	Description
Raspberry Pi	3B+	Main processing board
GrovePi+	v3.0	Sensors integration board
GPS module	v1.2	Retrieves latitude and longitude
Display module	v1.0	Displays the number of detected EI
Temperature sensor	v1.2	Temperature from $-40^{\circ }$ to $125^{\circ }$
Humidity sensor	v1.2	Relative humidity from 20% to 90%
Audio sensor	v1.6	From 0 a 1023; can be converted to dB
Water sensor	v1.1	Two states: 1 (dry) and 0 (wet)
Air Quality sensor	v1.2	From 0 to 1000
